# Disseminated *Aspergillus flavus* infection presenting as spondylodiscitis: a fatal tuberculosis mimicker in an immunocompetent host – case report and literature review

**DOI:** 10.3389/fmed.2026.1941883

**Published:** 2026-09-24

**Authors:** Olfa Smaoui, Helmi Ernandes, Fatma Gassara, Houda Limam, Imen Beji, Lamia Ammari, Aida Berriche

**Affiliations:** 1 Infectious Diseases Department, La Rabta Hospital, Tunis, Tunisia; 2 Infectious Diseases Department, Mohamed Kassab Institute of Orthopedics, Manouba, Tunisia

**Keywords:** antifungal drug resistance, *Aspergillus flavus*, invasive aspergillosis, spondylodiscitis, tuberculosis, voriconazole

## Abstract

**Introduction:**

Disseminated invasive aspergillosis is a rare but life-threatening fungal infection that predominantly affects immunocompromised individuals. Vertebral involvement is an uncommon manifestation, and diagnosis in immunocompetent patients is particularly challenging because of its nonspecific clinical and radiological features, often resulting in delayed diagnosis and inappropriate therapy. We report a fatal case of disseminated *Aspergillus flavus* aspergillosis presenting as spondylodiscitis in an immunocompetent host, highlighting the diagnostic challenges and therapeutic implications.

**Case description:**

A 54-year-old man with no recognized immunosuppressive condition presented with a five-month history of progressive dorsolumbar pain complicated by paraparesis. Magnetic resonance imaging demonstrated thoracic spondylodiscitis with extensive epidural and paravertebral abscesses. Histopathological examination of the discovertebral biopsy initially suggested tuberculous spondylodiscitis, leading to empirical antituberculous therapy and corticosteroid administration. Mycological culture and periodic acid–Schiff staining subsequently identified an *Aspergillus* section *Flavi* isolate phenotypically identified as *Aspergillus flavus*, establishing the diagnosis of *Aspergillus* spondylodiscitis. Empirical antifungal therapy with amphotericin B deoxycholate was initiated and later switched to voriconazole after antifungal susceptibility testing showed reduced *in vitro* susceptibility to amphotericin B and an elevated echinocandin minimum effective concentration, with preserved susceptibility to voriconazole. Additional investigations, including compatible cross-sectional imaging findings and a positive serum galactomannan antigen assay, supported presumed pulmonary and sphenoidal sinus involvement in addition to proven vertebral disease with extension to the adjacent ribs. Evaluation of host immunity revealed negative HIV serology and a preserved CD4 T-lymphocyte count (902 cells/µL), with no identifiable underlying risk factors. Despite targeted antifungal therapy, the patient experienced progressive neurological deterioration due to spinal cord compression, requiring emergency decompressive laminectomy. He subsequently developed postoperative septic shock and died.

**Conclusion:**

This case underscores that invasive aspergillosis should be considered in the differential diagnosis of chronic or subacute spondylodiscitis, even in apparently immunocompetent patients. Early diagnosis requires vertebral biopsy with dedicated fungal histopathology, mycological culture, species identification, and antifungal susceptibility testing to enable timely targeted therapy and early multidisciplinary management.

## Introduction

Invasive aspergillosis is a life-threatening fungal infection and the leading cause of mold-related mortality worldwide. It predominantly affects severely immunocompromised individuals, including recipients of hematopoietic stem cell transplantation, patients with prolonged neutropenia, and those receiving high-dose corticosteroid therapy (1). Disseminated invasive aspergillosis, defined as infection involving at least two noncontiguous organ systems, is an uncommon but particularly severe manifestation associated with mortality rates approaching 30% (2, 3). Consequently, disseminated disease in immunocompetent hosts remains an exceptional clinical entity that is poorly represented in current risk-stratification models and frequently associated with delayed or incorrect diagnosis (4, 5).

Among the more than 250 recognized *Aspergillus* species, *Aspergillus flavus* is the second most clinically significant pathogen after *A. fumigatus*, accounting for a substantial proportion of invasive infections worldwide (6–8). Its prevalence is particularly high in tropical and subtropical regions, where it represents a major cause of invasive fungal sinusitis (7). Importantly, *A. flavus* exhibits a greater propensity for osteotropic dissemination than *A. fumigatus*, with vertebral involvement constituting its most frequent skeletal manifestation (8–10). This distinctive tropism highlights the importance of accurate species identification because of its therapeutic and prognostic implications (7). Despite being the most common form of skeletal aspergillosis, vertebral aspergillosis remains exceedingly uncommon, with the literature consisting predominantly of isolated case reports and small case series (7, 11, 12).

Infection typically results from hematogenous dissemination following pulmonary or sinonasal disease. A hallmark of invasive aspergillosis is angioinvasion, whereby fungal hyphae penetrate vascular structures, causing thrombosis, tissue infarction, and dissemination to distant organs, including the spine, brain, and visceral organs (13). Disease progression is further facilitated by multiple virulence mechanisms, including extracellular enzyme production, biofilm formation, oxidative stress resistance, immune evasion, and mycotoxin biosynthesis (14–18).

Despite advances in understanding the molecular biology of *A. flavus*, important clinical challenges remain unresolved. The pathophysiology, diagnosis, and optimal management of vertebral aspergillosis in immunocompetent individuals are poorly characterized because available evidence is derived almost exclusively from immunocompromised populations (4, 5, 19). Radiological findings are often nonspecific and may closely resemble tuberculous spondylodiscitis, resulting in substantial diagnostic delays (19, 20). Furthermore, the performance of contemporary fungal biomarkers, including galactomannan, *β*-D-glucan, polymerase chain reaction (PCR), and lateral flow assays, has not been systematically evaluated in vertebral disease (21).

Management is further complicated by the absence of standardized therapeutic protocols. Recommendations regarding antifungal therapy, treatment duration, and surgical indications are largely extrapolated from guidelines for pulmonary aspergillosis because randomized studies in spinal disease are lacking (10). In parallel, the emergence of antifungal resistance driven by environmental and treatment-associated mutations poses a growing threat to treatment efficacy, with multidrug-resistant isolates increasingly reported across all major antifungal classes (22–24).

We report a case of disseminated *Aspergillus flavus* infection involving the paranasal sinuses, lungs, and thoracic spine, presenting as thoracic spondylodiscitis in a previously immunocompetent patient. This case underscores the diagnostic challenges of invasive vertebral aspergillosis in the absence of classical immunosuppressive risk factors, critically appraises the diagnostic workup in the context of current evidence, and compares our findings with previously reported cases in immunocompetent hosts.

## Case description

A 54-year-old male patient, with no recognized immunosuppressive condition, presented with progressively worsening dorsolumbar back pain over five months. His medical history was unremarkable for tobacco use, chronic pulmonary disease, occupational or environmental fungal exposure, recent viral respiratory infection, or prior immunosuppressive therapy. Physical examination revealed localized thoracolumbar spinal tenderness. The pain exhibited a mixed diurnal rhythm and gradually intensified, without associated fever, weight loss, or general health deterioration. The patient had sought medical attention several times and received symptomatic treatment only, without clinical improvement. The pain eventually became severe, resulting in marked functional limitation that rendered both standing and walking impossible.

On admission, the patient was afebrile and hemodynamically stable. Neurological examination showed paraparesis, with symmetric lower-limb weakness (Medical Research Council grade 3/5), while light-touch, pinprick, and proprioceptive sensation were preserved. Bladder and bowel function remained intact, corresponding to an American Spinal Injury Association Impairment Scale grade D. No other focal neurological deficits were identified. Cardiopulmonary, abdominal, and cutaneous examinations were unremarkable, with no clinical evidence of systemic infection. Initial laboratory investigations demonstrated leukocytosis (12.7 ×  10⁹/L) and an elevated C-reactive protein (CRP) concentration of 23.5 mg/L.

Plain radiography of the spine revealed disc space narrowing at the T9–T10 level, with a fusiform paravertebral opacity. Magnetic resonance imaging (MRI) demonstrated spondylodiscitis at T9–T10, associated with paravertebral abscesses, involvement of the posterior elements, and epidural fluid collections.

Given the subacute clinical evolution and radiological features, tuberculous spondylodiscitis was initially suspected. Brucellar spondylodiscitis was also considered as a differential diagnosis, owing to the endemic nature of brucellosis and the chronic course of symptoms. Pyogenic bacterial spondylodiscitis remained a possible differential diagnosis, though considered less likely given the absence of fever, the chronic progression, and negative initial bacteriology. Neoplastic processes, such as vertebral metastasis or lymphoma, were also considered in the differential but were ruled out based on the MRI morphology showing disc involvement and abscess formation, and the absence of evidence for a primary malignancy. Tuberculin skin testing, blood cultures, Rose Bengal testing, and Wright's serology were all negative.

Given these inconclusive noninvasive investigations, a disco-vertebral biopsy was performed on day 3, yielding multiple bone and soft tissue fragments for bacteriological, mycological, and histopathological examination, along with five cytological smears. Histopathological analysis revealed extensive necrotizing, granulomatous, and suppurative inflammation of the cancellous bone and periosteal tissue, with associated osseous sequestra.

Despite negative bacteriological investigations, including the absence of pyogenic bacteria and acid-fast bacilli, empirical antituberculous therapy comprising isoniazid, rifampicin, ethambutol, and pyrazinamide was initiated on hospital day 11 based on histopathological findings suggestive of tuberculosis and the regional endemicity of the disease. Adjunctive dexamethasone (0.4 mg/kg/day) was administered to reduce inflammatory spinal cord edema, and spinal immobilization with a Neofract corset was implemented for stabilization and pain control. The clinical course was subsequently complicated by corticosteroid-induced diabetes mellitus.

On the 6th day of therapy (hospital day 16), direct microscopic examination of the disco-vertebral biopsy specimen demonstrated septate fungal hyphae, while mycological culture yielded an *Aspergillus* section *Flavi* isolate phenotypically identified as *Aspergillus flavus*. Species-level identification was based on characteristic macroscopic and microscopic features, including yellow-green to olive-green colonies with a yellow to orange-brown reverse and a velvety to floccose texture. Microscopic examination revealed biseriate conidial heads with rough-walled conidiophores and finely to moderately echinulate, globose to subglobose conidia measuring approximately 3–6 µm, consistent with the morphological characteristics of *A. flavus*. Although molecular approaches or MALDI-TOF mass spectrometry provide greater discriminatory power for species within *Aspergillus* section *Flavi*, these techniques were not available in our laboratory. Reevaluation of the histological slides with periodic acid–Schiff (PAS) staining confirmed abundant septate hyphae and spores, establishing the diagnosis of *Aspergillus* spondylodiscitis.

Given the confirmed fungal etiology, anti-tuberculous therapy and corticosteroids were discontinued. Antifungal treatment with amphotericin B deoxycholate (1 mg/kg/day) was initiated while awaiting antifungal susceptibility testing. Amphotericin B was selected because of its broad-spectrum fungicidal activity, its recognized role as an alternative first-line agent for invasive aspergillosis, and the unavailability of voriconazole at the time of diagnosis. On hospital day 23, antifungal susceptibility results became available from testing by the Etest gradient diffusion method (bioMérieux) and were confirmed using the European Committee on Antimicrobial Susceptibility Testing (EUCAST) reference broth microdilution method (E.Def 9.4). Minimum inhibitory concentrations (MICs) and minimum effective concentrations (MECs) were interpreted according to EUCAST clinical breakpoints and epidemiological cutoff values (ECOFFs), when available, as described in Version 6.0 (2025). The isolate showed a markedly elevated amphotericin B MIC of 8 µg/mL by Etest and EUCAST broth microdilution, exceeding the *Aspergillus flavus* ECOFF of 4 µg/mL and indicating a non-wild-type phenotype. The caspofungin MEC was >16 µg/mL by both methods; no EUCAST ECOFF or clinical breakpoint is currently established for caspofungin against *A. flavus*. Voriconazole MICs were 0.38 µg/mL by Etest and 0.5 µg/mL by EUCAST, while posaconazole MICs were 0.064 and 0.06 µg/mL, respectively; both remained within the corresponding *A. flavus* wild-type distributions (ECOFFs, 2 and 0.5 µg/mL, respectively). Accordingly, antifungal therapy was switched to intravenous voriconazole, administered as a loading dose of 6 mg/kg every 12 h during the first 24 h, followed by a maintenance dose of 4 mg/kg every 12 h. On day 5 of therapy, therapeutic drug monitoring showed a voriconazole plasma trough concentration of 4.5 mg/L, within the target range of 2–6 mg/L recommended for severe or disseminated aspergillosis. Serial monitoring of liver function tests remained unremarkable throughout treatment, with no clinically significant treatment-related adverse events, including hepatotoxicity, visual disturbances, or neurotoxicity.

Following the identification of *Aspergillus flavus*, a comprehensive dissemination work-up was performed. Serological testing using the Platelia *Aspergillus* IgG enzyme immunoassay (Bio-Rad Laboratories, Marnes-la-Coquette, France) was positive, with an *Aspergillus*-specific IgG level of 22 UA/mL (positivity cutoff >10 UA/mL). Serum galactomannan antigen testing (Platelia *Aspergillus* Ag, Bio-Rad Laboratories) was also positive, with an optical density index (ODI) of 1.8 (positivity cutoff ≥0.5). Craniofacial, thoracic, and abdominopelvic computed tomography (CT) scans revealed a partially calcified nodular lesion of the sphenoidal sinus, multiple bilateral pulmonary nodules involving all lobes, findings consistent with angioinvasive pulmonary aspergillosis, and destructive vertebral lesions extending from T7 to T11, with involvement of the posterior elements and adjacent ribs. Extensive epidural, prevertebral, and paravertebral abscesses were observed, extending longitudinally from C3–C4 to L3–L4. A comprehensive ophthalmological examination showed no evidence of ocular involvement.

A comprehensive evaluation for underlying immunodeficiency and other predisposing conditions revealed no significant abnormalities. HIV serology was negative, and cellular immunity was preserved, with a CD4 T-lymphocyte count of 902 cells/µL (reference range: 500–1,500 cells/µL). Complete blood count revealed an absolute neutrophil count of 8.9 ×  10⁹/L (1.5–7.5 × 10⁹/L), and an absolute lymphocyte count of 2.1 × 10⁹/L (1.0–4.0 × 10⁹/L). Peripheral blood smear showed no morphological abnormalities. Baseline fasting plasma glucose was 5.1 mmol/L (reference range: 3.9–5.5 mmol/L), and glycated hemoglobin (HbA1c) was 5.4% (reference range: <5.7%). Serum protein electrophoresis was normal, antinuclear antibodies were negative, and serum immunoglobulin concentrations were within the reference ranges (IgG: 11.8 g/L [7.0–16.0 g/L], IgA: 2.3 g/L [0.7–4.0 g/L], and IgM: 1.2 g/L [0.4–2.3 g/L]). Collectively, these findings revealed no evidence of an underlying acquired immunodeficiency or other recognized predisposing condition for invasive aspergillosis.

A final diagnosis of disseminated invasive aspergillosis due to *Aspergillus flavus* was established, with proven vertebral involvement based on histopathological and mycological confirmation from the vertebral lesion and presumed pulmonary and sphenoidal sinus involvement based on compatible cross-sectional imaging findings, and a positive serum galactomannan antigen assay. Bronchoalveolar lavage, respiratory culture, *Aspergillus* PCR, and sinus endoscopy with biopsy were not performed; therefore, microbiological confirmation of these extraspinal sites was not available.

On the 15th day of voriconazole therapy (hospital day 37), the patient developed progressive neurological decline. An urgent spinal MRI (Figure 1) revealed extensive spondylodiscitis involving T7–T10, with destruction of the T7–T8, T8–T9, and T9–T10 intervertebral discs, involvement of the posterior vertebral elements, multiple intraosseous abscesses, anterior and posterior epidural abscesses, extensive prevertebral and paravertebral collections, and vertebral collapse resulting in focal kyphotic deformity. These findings were associated with severe spinal cord compression and compressive myelopathy.

**Figure 1:**
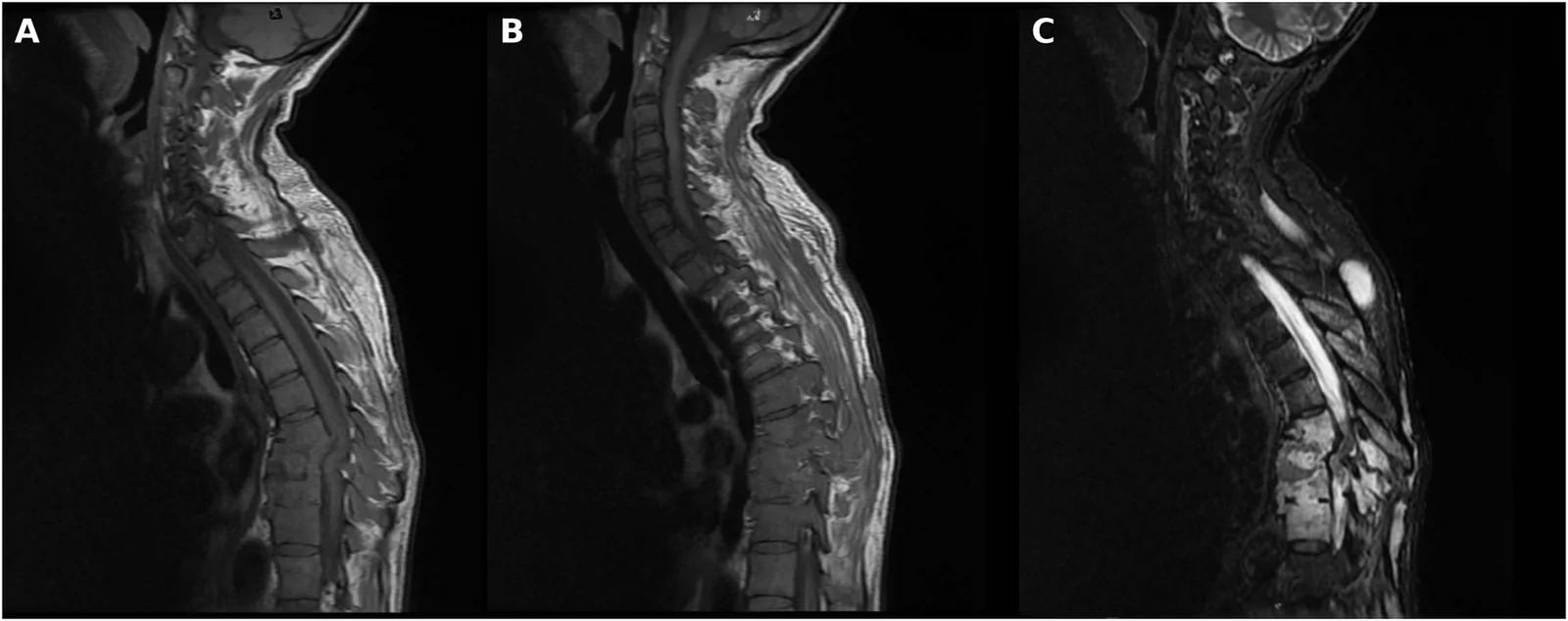
Magnetic resonance imaging (MRI) of the thoracic spine demonstrating extensive thoracic spondylodiscitis with epidural, paravertebral, and spinal cord involvement. **(A)** Sagittal T1-weighted image showing extensive spondylodiscitis involving the T7–T10 vertebral levels, characterized by diffuse hypointense marrow signal, destruction of the T7–T8, T8–T9, and T9–T10 intervertebral discs, partial collapse of the affected vertebral bodies, and extension to the posterior vertebral elements. **(B)** Sagittal contrast-enhanced fat-suppressed T1-weighted image demonstrating diffuse enhancement of the involved vertebral bodies and intervertebral discs, multiple intraosseous abscesses, anterior and posterior epidural collections, and extensive prevertebral and paravertebral inflammatory extension. **(C)** Sagittal short tau inversion recovery (STIR) image showing marked bone marrow and surrounding soft-tissue edema, multiloculated prevertebral and posterior paravertebral fluid collections, focal kyphotic angulation secondary to vertebral body collapse, and severe spinal cord compression with intramedullary hyperintensity extending from T7 to T9, consistent with compressive myelopathy.

Although emergency decompressive surgery was indicated, the procedure was initially deferred following anesthesiology assessment, which deemed the patient to be at very high perioperative risk and recommended reassessment under antifungal therapy. This approach aimed to optimize the patient's clinical condition and surgical candidacy before proceeding with major spinal surgery, following multidisciplinary consideration of the competing risks associated with persistent neural compression and emergency surgery in the setting of critical illness. Owing to continued neurological deterioration despite targeted antifungal treatment, emergency decompressive surgery was ultimately performed on hospital day 46, consisting of a T7 laminectomy with evacuation and drainage of thoracic and lumbar paravertebral abscesses, yielding approximately 700 mL of thick greenish purulent material. The T7 level was selected for laminectomy based on MRI findings demonstrating maximal spinal cord compression and associated intramedullary signal abnormality. In view of the patient's prohibitive perioperative risk, a focused decompressive approach was undertaken rather than multilevel decompression of all radiologically involved levels. Bacteriological examination of the surgical specimen yielded no bacterial growth, whereas mycological examination confirmed *Aspergillus flavus*, and antifungal susceptibility testing demonstrated an identical susceptibility profile to that of the initial disco-vertebral isolate. Despite aggressive postoperative intensive care, the patient experienced rapid clinical deterioration, characterized by refractory hypotension (mean arterial pressure <65 mmHg) despite adequate fluid resuscitation, necessitating escalating doses of norepinephrine, progressive hyperlactatemia (peak serum lactate, 6.8 mmol/L), and evolving multiorgan dysfunction, including acute kidney injury and coagulopathy. Blood cultures remained sterile. The patient died from refractory septic shock within 24 h of surgery, meeting the Sepsis-3 clinical criteria. Table 1 chronologically summarizes the patient's major symptoms, diagnostic findings, interventions, and outcomes.

**Table 1:** Timeline of the patient's clinical presentation, diagnostic work-up, and management.

Time point	Clinical Event	Diagnostic Findings	Management	Outcome
Month −5 to Day 0	Progressive dorsolumbar back pain; absence of fever, weight loss, or general deterioration	No etiological investigations performed	Symptomatic outpatient treatment only	Progressive worsening with severe functional limitation
Day 0 (Admission)	Severe back pain; inability to stand or walk; paraparesis	Leukocytosis: 12,700/mm^3^; CRP 23.5 mg/L	Hospital admission	Established neurological deficit
Day 0–Day 1	Initial spinal evaluation	Plain spinal radiography: T9–T10 disc space narrowing with fusiform paravertebral opacity	Diagnostic work-up initiated	Infectious spondylodiscitis diagnosis
	Advanced imaging assessment	MRI: T9–T10 spondylodiscitis with paravertebral abscesses, posterior element involvement, and epidural collections		
Day 1–3	Etiological investigation	TST, blood cultures, Rose Bengal, Wright serology negative; disco-vertebral biopsy performed on hospital day 3	Biopsy samples sent for bacteriological, mycological, and histopathological analysis	Awaiting definitive etiologic diagnosis
Day 11	Presumptive diagnosis of tuberculous spondylodiscitis	Histopathology: necrotizing granulomatous and suppurative inflammation	Empirical AT therapy, adjunctive dexamethasone and spinal immobilization	No clinical response
Day 16	Revision of etiological diagnosis	Direct microscopy and mycological culture: septate hyphae identified as *Aspergillus flavus* Histological confirmation of Aspergillosis	AT therapy and corticosteroids discontinued Initiation of AFT: AmB	*Aspergillus* spondylodiscitis diagnosis
Day 16–23	Assessment of disease dissemination	CT scans: sphenoidal sinus lesion, bilateral pulmonary nodules, T7–T11 vertebral involvement with extensive epidural, prevertebral, and paravertebral abscesses	Continuation of AFT	Diagnosis of disseminated IA involving the lungs, sphenoidal sinus, and spine
Day 23	Antifungal susceptibility testing	Resistance to AmB and caspofungin; susceptibility to voriconazole and posaconazole	Switch to voriconazole	Transient clinical stabilization
Day 37	Neurological deterioration	MRI: extensive T7–T10 spondylodiscitis with vertebral angulation and cord compression	Indication for urgent surgical intervention	Progressive neurological compromise
Day 46	Surgical management	Intraoperative findings: drainage of approximately 700 mL of purulent paravertebral collections	T7 laminectomy with abscess drainage	Immediate postoperative deterioration
< 24 h postoperatively	Hemodynamic collapse	Clinical diagnosis of septic shock	Intensive care management	Death

AmB, Amphotericin B; AT, Anti-tuberculous therapy; CRP, C-reactive protein; CT, Computed tomography; IA, Invasive aspergillosis; MRI, Magnetic resonance imaging; TST, Tuberculin skin test.

## Discussion

Disseminated aspergillosis with vertebral involvement is among the rarest and most therapeutically challenging manifestations of invasive aspergillosis. The present case is remarkable for several reasons, including the occurrence of disseminated *Aspergillus flavus* infection in a previously healthy immunocompetent adult without identifiable predisposing factors, the reduced *in vitro* susceptibility to amphotericin B and elevated echinocandin MEC observed on antifungal susceptibility testing, and a fatal outcome despite susceptibility-guided antifungal therapy and surgical intervention. These features raise important questions regarding disease pathogenesis, diagnosis, and management.

Invasive aspergillosis is traditionally regarded as a disease of immunocompromised hosts, and current risk stratification models are largely based on this assumption. However, our patient lacked recognized predisposing conditions, including prolonged neutropenia, chronic corticosteroid exposure, immunosuppressive therapy, cytomegalovirus infection, and graft-versus-host disease (25). Furthermore, an extensive evaluation revealed negative HIV serology, preserved cellular immunity, normal hematological and immunological parameters, and no evidence of diabetes mellitus or autoimmune disease. Despite the absence of identifiable host-related risk factors, the patient developed extensive hematogenous dissemination involving the sphenoidal sinus, lungs, and spine.

This apparent contradiction has been increasingly reported in recent literature. Growing evidence suggests that susceptibility to invasive aspergillosis may result from subtle or unrecognized immune abnormalities that are not detected by routine immunological investigations. These include functional neutrophil defects, complement deficiencies, genetic variants affecting pattern-recognition receptors such as toll-like receptors and dectin-1, as well as transient immune dysregulation associated with occult viral infections (26). In addition, severe respiratory viral infections, particularly influenza and COVID-19, have emerged as important risk factors for invasive pulmonary aspergillosis in patients without classical immunosuppression through disruption of mucosal barriers and impairment of innate immune defenses (27). Although no such predisposing factor could be identified in our patient, the possibility of an underlying immunogenetic susceptibility or unrecognized transient immune dysfunction cannot be excluded. These observations emphasize that invasive aspergillosis should remain a diagnostic consideration even in apparently immunocompetent individuals.

Another notable aspect of this case is the absence of preceding trauma, spinal instrumentation, or other identifiable iatrogenic exposure. In immunocompetent patients, most reported cases of vertebral aspergillosis have been associated with direct inoculation following invasive procedures or traumatic injury (9, 20). By contrast, the coexistence of bilateral pulmonary nodules and positive fungal serological markers in our patient strongly supports hematogenous dissemination from a primary pulmonary focus. This mode of infection, although biologically plausible, remains uncommon and further illustrates the limitations of conventional risk-based assumptions regarding vertebral aspergillosis.

More broadly, the pathogenesis of disseminated *Aspergillus flavus* infection is driven by a complex interplay between fungal virulence mechanisms and host immune responses. As with other *Aspergillus* species, the lungs constitute the principal portal of entry owing to the inhalational route of spore acquisition and the organism's marked aerophilic tropism (6). Following pulmonary colonization and tissue invasion, angioinvasion, the penetration of vascular endothelium by fungal hyphae, facilitates hematogenous dissemination to distant organs and represents the key pathogenic mechanism underlying extrapulmonary disease (28). Disseminated infection may subsequently involve multiple sites, including the eye, central nervous system, heart, sinus, and skeletal system (4, 5). Among osseous manifestations, vertebral involvement is the most frequently reported localization, followed by long-bone and joint infections (1, 10, 29).

The marked propensity of *A. flavus* for skeletal dissemination is well documented and may be explained by several species-specific biological characteristics (8–10). Once bloodstream dissemination occurs, the osseous microenvironment appears to provide a favorable niche for *A. flavus*. Distinct proteolytic profiles, adhesion-related factors, and metabolic adaptations may confer enhanced survival and persistence within bone tissue compared with *A. fumigatus* (7, 14). This biological predisposition may account for the disproportionately high frequency of *A. flavus* reported in vertebral aspergillosis relative to its overall contribution to invasive aspergillosis cases (8–10).

In addition to its propensity for skeletal involvement, *A. flavus* is increasingly recognized as a major cause of fungal sinusitis and keratitis, particularly in tropical and subtropical regions (7, 8). Epidemiological studies have identified *A. flavus* as the predominant cause of fungal sinusitis across much of Asia and Africa, although its geographic distribution varies, with a lower prevalence reported in North America (7).

At the molecular level, pathogenicity is orchestrated through a coordinated network of virulence determinants. Prior studies have identified regulatory pathways involving *rtfA*, *creA*, and *aflS* that enhance extracellular matrix degradation, tissue colonization, and fungal persistence within host tissues (14, 15, 30). Dissemination is further facilitated by immune-evasion mechanisms, including resistance to oxidative killing, modulation of *β*-glucan exposure, secretion of immunomodulatory factors, and biofilm formation (17, 18, 31). Ultimately, disease progression depends on the balance between fungal virulence and host immune responses, particularly Th1/interferon-*γ*-mediated antifungal immunity (17).

The clinical course of vertebral aspergillosis further contributes to delayed diagnosis. Symptoms are typically insidious, with progressive axial pain evolving over weeks or months and neurological deficits appearing only in advanced stages (9, 20). Our patient exemplified this pattern, experiencing progressive dorsolumbar pain for five months before neurological deterioration prompted hospitalization. By the time of diagnosis, dissemination to multiple organ systems had already occurred, highlighting how the indolent nature of the disease can contribute to advanced presentation and poor outcomes. This delay mirrors previous observations, with a systematic review reporting a mean interval of 5.7 months between symptom onset and the diagnosis of *Aspergillus* spondylodiscitis (32).

This diagnostic delay is further compounded by the limited specificity of radiological findings. In pulmonary disease, computed tomography may demonstrate characteristic findings such as the halo sign during early infection and the air-crescent sign during later stages (33). However, significant overlap exists between pulmonary tuberculosis and aspergillosis, as both conditions may present with cavitary lesions and nodular infiltrates, often precluding reliable differentiation on imaging alone (34–36). Similarly, the radiological features of *Aspergillus* spondylodiscitis, including vertebral destruction, disc-space narrowing, and paravertebral soft-tissue collections, are largely indistinguishable from those observed in tuberculous spondylitis on CT and MRI (19, 36). Maxillary sinus involvement is classically reported as the most frequent localization of *Aspergillus* sinusitis, whereas isolated sphenoidal disease, as observed in our case, remains exceptional (37). In addition, radiological findings such as sinus calcifications, which were present in our patient, and osteolytic changes are considered highly suggestive of an *Aspergillus* etiology (37). Nevertheless, these features are not routinely sought during the investigation of vertebral infections.

Histopathological examination remains the cornerstone of diagnosis and is generally regarded as the diagnostic gold standard (21). However, interpretation may be challenging because fungal and tuberculous spondylodiscitis can share similar pathological features, particularly necrotizing granulomatous inflammation (38). This overlap contributed directly to the initial diagnostic orientation in our patient, who was empirically treated for tuberculosis before fungal infection was recognized. Definitive diagnosis requires demonstration of septate, acute-angle branching hyphae compatible with *Aspergillus* species using specific fungal stains such as periodic acid–Schiff (PAS) and Grocott methenamine silver (GMS) (21, 39). Importantly, histopathological findings should always be complemented by mycological culture, which remains essential for species identification and antifungal susceptibility testing (21). The latter is particularly critical in the current era of emerging antifungal resistance, where therapeutic decisions increasingly depend on microbiological characterization (40).

Recent advances have expanded the diagnostic armamentarium available for invasive aspergillosis. Contemporary diagnostic algorithms incorporate serum and bronchoalveolar lavage galactomannan testing, serum (1→3)-β-D-glucan assays, PCR-based detection of *Aspergillus* DNA with simultaneous screening for resistance-associated mutations, and point-of-care lateral flow immunoassays (21). Both galactomannan detection and PCR-based assays have been incorporated into the ESCMID–ECMM–ERS diagnostic criteria for invasive aspergillosis (21). In our patient, a markedly positive galactomannan level, obtained retrospectively after mycological confirmation, suggests that earlier implementation of fungal biomarker testing might have accelerated diagnosis.

Taken together, these diagnostic challenges highlight the need to systematically consider fungal etiologies in the differential diagnosis of spondylodiscitis, particularly when clinical, radiological, or microbiological features are atypical for tuberculosis or when patients fail to improve despite empirical antituberculous treatment. Comprehensive diagnostic evaluation should include histopathological examination with dedicated fungal staining, mycological cultures, and, whenever available, fungal biomarker testing. Such an approach may reduce diagnostic delays, avoid inappropriate treatment, and facilitate earlier initiation of targeted antifungal therapy.

Beyond diagnosis, the clinical course of this case was profoundly shaped by antifungal resistance. The antifungal susceptibility profile observed in our case, reduced *in vitro* susceptibility to amphotericin B (MIC 8 µg/mL) and caspofungin (MEC >16 µg/mL), with susceptibility retained only to voriconazole and posaconazole, substantially restricted available therapeutic options. The epidemiology of antifungal resistance in *Aspergillus* is shaped by both environmentally acquired and treatment-associated mechanisms (24, 40). *Aspergillus flavus* is typically susceptible to azoles, including voriconazole, itraconazole, and posaconazole, as well as to echinocandins. In contrast, susceptibility to amphotericin B is more variable, with some isolates exhibiting reduced susceptibility despite the absence of absolute intrinsic resistance (40).

Although our isolate remained susceptible to voriconazole and posaconazole, azole resistance in *A. flavus* has emerged as a significant global concern. Resistant isolates have been reported across Asia, Europe, the Americas, and Africa, including China, India, Korea, Vietnam, Taiwan, Iran, Spain, the Netherlands, Italy, France, Brazil, Argentina, Sudan, and Tunisia, highlighting the worldwide distribution of resistance and the need for systematic surveillance in clinical practice (40). Azole resistance is primarily mediated by alterations of the *Cyp51A* target enzyme. Among these, the Y119F substitution in *A. flavus*, homologous to Y121F in *A. fumigatus*, has been associated with the emergence of resistance during prolonged antifungal therapy (22, 40). Additional resistance mechanisms have also been described, contributing to multidrug-resistant phenotypes (22, 23).

Species belonging to *Aspergillus* section *Flavi* are known to exhibit elevated amphotericin B minimum inhibitory concentrations (MICs), although they are not generally considered intrinsically resistant. An important exception is *A. alliaceus*, which consistently demonstrates amphotericin B MICs ≥8 mg/L (40). In a literature review encompassing 3,663 *A. flavus* isolates, Fakhim et al. estimated amphotericin B resistance at 14.9%, with resistance rates of 14.3% in Europe and 11.7% in the United States (41). The elevated amphotericin B MIC observed in our isolate therefore represents an uncommon but well-documented resistance phenotype. Although the mechanisms underlying amphotericin B resistance remain incompletely understood, alterations in ergosterol biosynthesis and membrane composition have been implicated (40).

In contrast, data regarding echinocandin resistance in *A. flavus* remain comparatively limited. Nevertheless, elevated MICs have been reported in clinical isolates. In Iran, 6 of 14 clinical *A. flavus* isolates (42.9%) demonstrated MIC values ≥4 mg/L (40). The concurrent elevated caspofungin MEC observed in our isolate effectively eliminated echinocandin-based salvage or combination strategies. Although the mechanisms of echinocandin resistance remain incompletely understood, both target-site alterations and alternative adaptive pathways have been implicated (42).

Clinically, antifungal resistance is independently associated with increased mortality, treatment failure, and breakthrough infections during antifungal prophylaxis (24, 40). This case underscores the importance of routine antifungal susceptibility testing for all *Aspergillus* isolates recovered from normally sterile sites, as susceptibility profiles may differ considerably from expected species-specific patterns and directly influence therapeutic decision-making.

Management of disseminated aspergillosis remains particularly challenging and requires prompt initiation of effective antifungal therapy. Both the IDSA (Infectious Diseases Society of America) and ESCMID (European Society of Clinical Microbiology and Infectious Diseases) guidelines recommend voriconazole as first-line treatment for invasive, disseminated or vertebral aspergillosis based on its superior efficacy demonstrated in randomized clinical trials (3, 21). Historical experience has shown the efficacy of Amphotericin B formulations but there is little reported experience in the use of posaconazole or echinocandins in the treatment of *Aspergillus* osteomyelitis (3). In our patient, empirical amphotericin B was initiated while awaiting antifungal susceptibility results and access to voriconazole, a defensible strategy given its broad-spectrum fungicidal activity and the clinical urgency imposed by spinal cord compression. Following susceptibility testing, treatment was appropriately switched to voriconazole in accordance with current recommendations. However, the initial seven-day exposure to amphotericin B, an agent to which the isolate was subsequently shown to be resistant, delayed effective antifungal therapy. This scenario supports the need for accelerated or bedside antifungal susceptibility testing and raises the question of whether empirical voriconazole should be initiated in parallel in patients with suspected high-risk invasive mold infections while awaiting microbiological confirmation.

The optimal duration of antifungal therapy for disseminated aspergillosis remains uncertain. Although the IDSA guidelines recommend a minimum treatment duration of 6–8 weeks, with longer courses (>6 months) frequently necessary (3), this recommendation is based solely on expert opinion, without support from systematic literature reviews or prospective clinical studies. In the review by Gamaletsou et al. of *Aspergillus* osteomyelitis, the mean duration of antifungal therapy was approximately 90 days, although considerable heterogeneity was observed according to disease extent and host characteristics (43). Published case reports generally support substantially prolonged treatment courses. Nicolle et al. reported successful management of isolated vertebral *Aspergillus fumigatus* infection following 12 months of voriconazole (11), whereas Dong et al. described relapse of *A. versicolor* spondylitis after an initial 4-month treatment course, requiring at least 12 additional months of antifungal therapy (44). Current expert recommendations therefore advocate continuation of antifungal treatment until complete clinical, radiological, and microbiological resolution is achieved, with serial imaging and biomarker monitoring (3). Collectively, these observations emphasize that premature discontinuation of therapy, even in patients demonstrating an initial clinical response, carries a substantial risk of relapse.

The role of combination antifungal therapy remains incompletely defined. At present, there are no *in vitro* or *in vivo* data specifically evaluating combination regimens against azole-resistant non-*fumigatus Aspergillus* species, representing an important limitation in the evidence base (40). Consequently, therapeutic strategies must largely be extrapolated from studies involving azole-resistant *A. fumigatus* and wild-type non-*fumigatus Aspergillus* species. In *A. fumigatus*, combinations of azole with echinocandin have shown encouraging results, particularly in isolates exhibiting moderate azole resistance, suggesting that similar approaches may also be beneficial for non-*fumigatus* species (40). Likewise, several studies have reported either synergistic or indifferent interactions between azoles and echinocandins against wild-type non-*fumigatus Aspergillus* species (40). Nevertheless, the available clinical evidence remains limited. In a systematic review of *Aspergillus* osteomyelitis, combination antifungal therapy did not demonstrate an apparent advantage over monotherapy (43). Current guidelines therefore reserve combination antifungal therapy primarily for refractory or disseminated infections (3, 21, 45–47). In our patient, this strategy was not feasible because concomitant resistance to caspofungin precluded the use of an echinocandin-based regimen.

Surgical intervention remains an essential adjunct to medical therapy in selected patients. In the comprehensive review by Gamaletsou et al., spinal cord compression was documented in 47% of patients with vertebral *Aspergillus* osteomyelitis (43). When spinal cord or nerve root compression, spinal instability, or epidural abscess formation is present, conservative antifungal therapy alone is generally insufficient and may permit progressive neurological deterioration. Under these circumstances, combined surgical and antifungal management is recommended (21, 48). Emergency T7 decompressive laminectomy with drainage of approximately 700 mL of purulent material was therefore appropriately performed in our patient. Surgical debridement provides several theoretical benefits, including reduction of fungal burden, removal of poorly perfused necrotic tissue refractory to systemic antifungal penetration, decompression of neural structures, and acquisition of tissue for definitive microbiological diagnosis and antifungal susceptibility testing (3, 21, 43). Moreover, fewer relapses are reported among patients treated with surgery combined with antifungal therapy compared with antifungal therapy alone (43). The exceptionally large intraoperative purulent collection observed in our patient reflected the advanced stage of disease at the time of intervention, while the inability to survive the physiological stress of surgery underscored the severity of infection. The optimal timing of surgical intervention, ideally before disease progression precludes recovery, remains one of the major unresolved questions in the management of vertebral aspergillosis (10).

The role of adjunctive corticosteroid therapy in fungal spondylodiscitis represents a genuine clinical dilemma that is not addressed by current guidelines. Corticosteroids are generally contraindicated in invasive aspergillosis because of their immunosuppressive effects, which may facilitate fungal proliferation (3, 21). Furthermore, corticosteroid exposure is a recognized risk factor for invasive aspergillosis, including *Aspergillus* osteomyelitis (32, 43, 48, 49). In retrospect, the initial corticosteroid therapy administered for presumed tuberculous spondylodiscitis may have contributed to disease progression in our patient. The subsequent development of steroid-induced diabetes mellitus further illustrates the metabolic complications of corticosteroid therapy and its potential to further impair host immune defenses. Even in the setting of acute spinal cord compression, corticosteroid therapy should generally be avoided because of the risk of exacerbating invasive fungal infection. If considered absolutely unavoidable to alleviate severe neurological compromise, it should be administered at the lowest effective dose, for the shortest possible duration, and always in conjunction with concurrent effective antifungal therapy. This difficult therapeutic balance underscores the challenge of preserving neurological function while minimizing the risk of further fungal progression. Notably, a recent meta-analysis demonstrated significantly higher aspergillosis-related mortality among patients receiving corticosteroid doses exceeding 2 mg/kg/day prednisolone equivalents, further supporting a highly restrictive corticosteroid-use policy (50).

To identify comparable published cases, a structured PubMed/MEDLINE search was conducted for English- or French-language case reports and case series describing presumed hematogenous vertebral *Aspergillus* infection without preceding spinal surgery, trauma, or direct inoculation (epidural injections, or percutaneous biopsy) in patients without recognized immunosuppressive conditions. Potentially eligible reports were screened by title and abstract, followed by full-text assessment, with reference-list screening to identify additional cases. Reports lacking histopathological or mycological confirmation, involving exclusively pediatric patients or non-vertebral infection, or providing insufficient clinical detail were excluded. The identified cases are summarized in Table 2.

**Table 2:** Clinical characteristics, management, and outcomes of published cases of hematogenous *Aspergillus* spondylodiscitis in immunocompetent hosts compared with the present case.

Case	Age/Sex	Species	Sites of Involvement	Initial Diagnosis	Antifungal Therapy	Treatment Duration	Surgical Management	Outcome
1. Present case	54/M	*A. flavus*	Sphenoidal sinus, lungs, spine, and adjacent ribs	TB spondylodiscitis	AmB followed by voriconazole	29 days	T7 laminectomy with abscess drainage	Died of septic shock
2. (4)	66/M	*Aspergillus* spp.	Lungs, eye, and spine	NR	IV voriconazole, AmB, and itraconazole, followed by oral voriconazole	12 months	None	Recovered
3. (51)	53/F	*A. fumigatus*	Spondylodiscitis	NR	Fluconazole and AmB	2 months	No	Died
4. (52)	19/M	*Aspergillus* spp.	Spondylodiscitis	NR	Itraconazole	2 months	Yes	Recovered
5. (52)	25/M	*Aspergillus* spp*.*	Spondylodiscitis	TB spondylodiscitis	Itraconazole	3 months	Yes	Recovered
6. (44)	60/NR	*A. versicolor*	Spondylitis	TB spondylitis Multiple myeloma Metastatic disease	Voriconazole; initial 4-month course followed by a second course of ≥12 months after relapse	≥12 months	None	Recovered; no recurrence at follow-up
7. (11)	65/M	*A. flavus*	Cervical spondylodiscitis and sphenoid bone involvement	Pyogenic spondylodiscitis	Voriconazole	12 months	No	Recovered; no relapse after 18 months of follow-up
8. (12)	67/M	*A. terreus*	Spine, sternoclavicular joint, and sacroiliac joint	Pyogenic spondylodiscitis	Voriconazole	NR	No	NR
9. (53)	68/M	*A. fumigatus*	Lung and spine	NR	Voriconazole and isavuconazole	12 months	Yes	Recovered
10. (54)	Early 60s/F	*A. flavus*	Spondylodiscitis	Pyogenic infection	Posaconazole	12 months	Yes	Recovered
11. (55)	53/M	*Aspergillus* spp.	Lungs and spine	Pyogenic infection TB infection Nocardiosis	Voriconazole	3 months	Yes	Recovered

AmB, amphotericin B; F, female; IV, intravenous; M, male; NR, not reported; TB, tuberculosis.

The unfavorable outcome in our patient was likely the result of the convergence of several well-established adverse prognostic factors. The literature identifies delayed diagnosis and initiation of antifungal therapy, central nervous system or disseminated disease, antifungal resistance, and the inability to achieve adequate surgical source control as the principal determinants of mortality in invasive aspergillosis (32, 43, 56). Despite advances in diagnosis and treatment, mortality remains substantial, with a median mortality rate of approximately 25% in *Aspergillus* osteomyelitis and increasing to 30% in disseminated disease (3, 43). In our patient, several of these adverse prognostic factors coexisted, including: (i) a 23-day delay in the initiation of appropriate antifungal therapy due to initial treatment for presumed tuberculous spondylodiscitis and strain reduced *in vitro* susceptibility to amphotericin B; (ii) extensive multi-organ dissemination with advanced epidural and paravertebral involvement; (iii) corticosteroid-induced immunosuppression and subsequent steroid-induced diabetes mellitus, further compromising host defenses; and (iv) the need for surgical intervention at an advanced stage of disease, when the systemic fungal burden had likely become irreversible. The cumulative effect of these factors most likely contributed to the fatal outcome. From a broader perspective, this growing burden of antifungal resistance, discussed above, poses a continued threat to the successful management of invasive aspergillosis (24, 40). This challenge is particularly relevant for rare extrapulmonary manifestations such as vertebral aspergillosis, for which high-quality evidence remains limited (10). Consequently, there is an urgent need for rapid susceptibility testing, standardized susceptibility-guided therapeutic algorithms, optimized antifungal combination strategies, and the development of novel antifungal agents to improve outcomes in these complex infections (10, 57).

Several limitations should be considered when interpreting this case. Species-level identification relied exclusively on conventional phenotypic methods. Molecular sequencing and MALDI-TOF mass spectrometry, which provide greater discriminatory power among closely related species within *Aspergillus* section *Flavi*, were not available in our laboratory; therefore, misidentification of a cryptic or closely related *Flavi* species cannot be entirely excluded. Pulmonary and sphenoidal sinus involvement was presumed based on compatible cross-sectional imaging and positive serum galactomannan antigen testing, without site-specific microbiological or histopathological confirmation. Accordingly, microbiological and histopathological confirmation was limited to vertebral lesion.

Invasive aspergillosis, particularly when presenting as spondylodiscitis, remains a rare but potentially fatal infection characterized by diagnostic uncertainty, delayed recognition, and the emerging challenge of antifungal resistance. This case highlights several important clinical lessons. Foremost, the absence of conventional immunosuppressive risk factors should not preclude consideration of invasive aspergillosis in patients presenting with chronic or subacute spondylodiscitis, particularly when bacteriological investigations are inconclusive and empirical antibacterial or antituberculous therapy fails to produce clinical improvement. Early recognition requires a comprehensive diagnostic approach integrating microbiological, histopathological, and radiological investigations. Mycological cultures, dedicated fungal staining with periodic acid–Schiff and Grocott methenamine silver, accurate species-level identification, and antifungal susceptibility testing are essential for establishing the diagnosis promptly and guiding appropriate therapy. Optimal management further relies on early multidisciplinary collaboration among infectious diseases specialists, neurosurgeons, radiologists, pathologists, and medical mycologists, with timely surgical intervention when indicated and individualized prolonged antifungal therapy guided by clinical, radiological, microbiological, and biomarker responses. Until higher-quality evidence becomes available, heightened clinical suspicion, comprehensive diagnostic evaluation, susceptibility-guided antifungal therapy, and multidisciplinary management remain the cornerstones of improving outcomes in this uncommon but devastating infection.

## Data Availability

The original contributions presented in the study are included in the article/Supplementary Material, further inquiries can be directed to the corresponding author.
